# A Novel Prognostic Model for Oral Squamous Cell Carcinoma: The Functions and Prognostic Values of RNA-Binding Proteins

**DOI:** 10.3389/fonc.2021.592614

**Published:** 2021-07-30

**Authors:** Yingjuan Lu, Yongcong Yan, Bowen Li, Mo Liu, Yancan Liang, Yushan Ye, Weiqi Cheng, Jinsong Li, Jiuyang Jiao, Shaohai Chang

**Affiliations:** ^1^Guangdong Provincial Key Laboratory of Malignant Tumor Epigenetics and Gene Regulation, Sun Yat Sen Memorial Hospital, Sun Yat-Sen University, Guangzhou, China; ^2^Department of Oral and Maxillofacial Surgery, Sun Yat Sen Memorial Hospital, Sun Yat-Sen University, Guangzhou, China; ^3^RNA Biomedical Institute, Sun Yat Sen Memorial Hospital, Sun Yat-Sen University, Guangzhou, China; ^4^Department of Hepatobiliary Surgery, Sun Yat Sen Memorial Hospital, Sun Yat-Sen University, Guangzhou, China

**Keywords:** oral squamous cell carcinoma, RNA-binding proteins, prognostic model, bioinformatic tools, nomogram

## Abstract

**Purpose:**

The biological roles and clinical significance of RNA-binding proteins (RBPs) in oral squamous cell carcinoma (OSCC) are not fully understood. We investigated the prognostic value of RBPs in OSCC using several bioinformatic strategies.

**Materials and Methods:**

OSCC data were obtained from a public online database, the Limma R package was used to identify differentially expressed RBPs, and functional enrichment analysis was performed to elucidate the biological functions of the above RBPs in OSCC. We performed protein-protein interaction (PPI) network and Cox regression analyses to extract prognosis-related hub RBPs. Next, we established and validated a prognostic model based on the hub RBPs using Cox regression and risk score analyses.

**Results:**

We found that the differentially expressed RBPs were closely related to the defense response to viruses and multiple RNA processes. We identified 10 prognosis-related hub RBPs (ZC3H12D, OAS2, INTS10, ACO1, PCBP4, RNASE3, PTGES3L-AARSD1, RNASE13, DDX4, and PCF11) and effectively predicted the overall survival of OSCC patients. The area under the receiver operating characteristic (ROC) curve (AUC) of the risk score model was 0.781, suggesting that our model exhibited excellent prognostic performance. Finally, we built a nomogram integrating the 10 RBPs. The internal validation cohort results showed a reliable predictive capability of the nomogram for OSCC.

**Conclusion:**

We established a novel 10-RBP-based model for OSCC that could enable precise individual treatment and follow-up management strategies in the future.

## Introduction

Oral cancer, comprising a group of tumors located in the alveolar ridge, buccal cavity, mucosa, floor of the mouth, palate, tongue, and other parts of the oral cavity, accounts for an estimated 350,000 new cases and 170,000 deaths per year worldwide. Of these, oral squamous cell carcinoma (OSCC) accounts for more than 95% of oral tumors. A major portion of global OSCC cases are diagnosed in Asia (1). Despite developments in the therapeutic strategies of OSCC that have been achieved over the last few years, the long-term survival rates of OSCC patients are still extremely low. Additionally, precise targeted therapies remain limited, resulting in treatment failure due to individual genetic differences and epigenetic changes among OSCC patients (2–4). Thus, a systematic study to identify key biomarkers for diagnosis and effective targets for treatment of OSCC is urgently needed.

Nomograms are statistics-based tools that can effectively integrate different variables and are widely used to detect prognostic factors for various cancers (5, 6). Many models have been established to predict the outcomes of OSCC by calculating the risk associated with clinicopathologic characteristics, such as age, sex, smoking status, TNM staging, and therapies (7, 8). Although these studies have enhanced our understanding of OSCC, few have taken into account genetic features, and these studies are not sufficiently accurate and reliable, as the underlying molecular mechanisms of OSCC might be considerably complex (9).

RNA-binding proteins (RBPs) modulate the functions of RNA transcripts at multiple levels (10). Currently, 1,542 RBPs have been identified, and more than 800 mRNAs have been discovered in human cells (11, 12). These RBPs bind RNA through globular RNA-binding domains (RBDs) and affect the splicing, stability, or translation of their target RNAs (13, 14). Considering the importance of RBPs in many cellular processes, it is not surprising that defects in their functions affect several human diseases, including neurological disorders, muscular atrophy, and cancer (15). Many studies have indicated that the dysregulation of RBPs is related to the biological behaviors of various cancer cells. For instance, human antigen R (HuR) is widely overexpressed in cancer cells and involved in posttranscriptional gene regulation (16). HuR not only affects lncRNAs but also binds to protein-coding mRNAs, exerting both positive and negative influences on their stability (17). The abnormal expression of heterogeneous nuclear ribonucleoprotein K (hnRNPK) is frequently observed in cancers and might play an important role in hepatocellular carcinoma (HCC) by changing the localization of MALAT1 (18). Serine/arginine-rich splicing factor 1 (SRSF1) is an RBP that is upregulated in glioma and plays an important role by regulating splicing, RNA stability, and nuclear export (19). Insulin-like growth factor 2 mRNA-binding protein 1 (IGF2BP1) modulates oncogenic impact on regulating cell proliferation and apoptosis in HCC (20). Mitogen-activated protein kinase-activated protein kinase-2 (MAPKAPK2) regulates transcript stability and exerts important effects in head and neck squamous cell carcinoma (HNSCC) (21). In summary, these previous studies have shown that RBPs may play important roles in tumorigenesis and tumor progression. However, only a few RBPs have been studied recently, and some RBPs were revealed to have critical functions in cancers.

In this study, we integrated all relevant OSCC information from The Cancer Genome Atlas (TCGA) database to perform a novel systematic analysis. To reveal the potential functions and clinical significance of RBPs in the pathogenesis of OSCC, we screened multiple differentially expressed RBPs in OSCC. Some of them may provide potential biomarkers for OSCC patients’ prognosis prediction.

## Materials and Methods

### Dataset Collection and Identification of Differentially Expressed RBPs

The RNA sequencing data of 32 normal oral tissues and 331 OSCC tissues with corresponding clinical information were obtained from TCGA (https://portal.gdc.cancer.gov/). Approval of the institutional ethics committee was not necessary because the data were collected from publicly available online databases. To identify the differentially expressed genes between normal oral and OSCC tissue samples, all raw data were preprocessed using the Limma package. In addition, we also identified differentially expressed RBPs with a threshold of |log2-fold change (FC)| ≥1 and a false discovery rate (FDR) <0.05. All TCGA patients were randomly divided into a training cohort with N*p (p = 2/3) samples and an internal validation cohort with N*q (q = 1/3) samples by caret R package. To validate our results responsibly, we searched for external validation cohort from Sun Yat-sen Memorial Hospital (n=58). This study was approved by the institutional review board of Sun Yat-Sen Memorial Hospital, Sun Yat-Sen University.

### KEGG Pathway and GO Enrichment Analyses

The biological functions of differentially expressed RBPs were systematically detected by Gene Ontology (GO) enrichment and Kyoto Encyclopedia of Genes and Genomes (KEGG) pathway analyses. The GO analysis included terms in the cellular component (CC), molecular function (MF), and biological process (BP) categories. All enrichment analyses were conducted by the WEB-based Gene Set Analysis Toolkit (WebGestalt, http://www.webgestalt.org/) (22). For both analyses, P values less than 0.05 and gene numbers greater than 5 were considered statistically significant.

### PPI Network Construction and Module Screening

The protein-protein interactions (PPIs) among all differentially expressed RBPs were investigated using the Search Tool for the Retrieval of Interacting Genes/Proteins (STRING) database (http://www.string-db.org/) (23). Cytoscape 3.8.0 software was used to further visualize the PPI network. The Molecular Complex Detection (MCODE) plug-in was used to select key modules and genes in the PPI network with both MCODE scores and node count numbers greater than 5 (24). A P value <0.05 was considered to indicate a significant difference.

### Prognosis-Related RBP Identification and Prognostic Model Construction

We performed univariate Cox regression analysis on the differentially expressed RBPs in the critical modules of the training dataset using the survival R package. Subsequently, we performed a log-rank test to extract prognosis-related RBPs. Based on the above preliminarily screened significant candidate RBPs, a multivariate Cox proportional hazards regression model was further performed, and the risk score (RS) was calculated to predict the prognostic outcomes of OSCC patients (25). The RS formula for each sample was as follows: RS=Expressiongene1×βgene1+ ··· + Expressiongenen×βgenen (where β is the regression coefficient derived from multivariate Cox regression). OSCC patients were divided into low-risk and high-risk groups according to the median RS analysis. The log-rank test was used to compare the difference in overall survival (OS) between the two groups. In addition, the survival ROC package was used to assess the prognostic capability of the above model (26). In addition, an internal validation cohort was used to test the predictive capability of the nomogram. Calibration curves were constructed using the rms R package to observe the accuracy of the prediction. A P value <0.05 indicated a significant difference.

### Verification of Express Level and Prognostic Significance by Immunohistochemistry (IHC)

Fixed OSCC and adjacent normal tissues samples were sectioned at 4 µm thickness. The tissue samples were deparaffinized, subjected to antigen retrieval with sodium citrate, and incubated with 3% hydrogen peroxide for 15 min at room temperature. Then, they were blocked with goat serum for 15 min at 37°C and incubated with the indicated antibody at 4°C overnight. Subsequently, the secondary antibody was added and incubated for 1 h at room temperature. Finally, the sections were visualized with 3,3’-diaminobenzidine. The stained slides were scanned, and the images were then digitalized and analyzed using Image-Pro Plus 5.1 software.

## Results

### Identification of Differentially Expressed RBPs in OSCC

**Figure 1:**
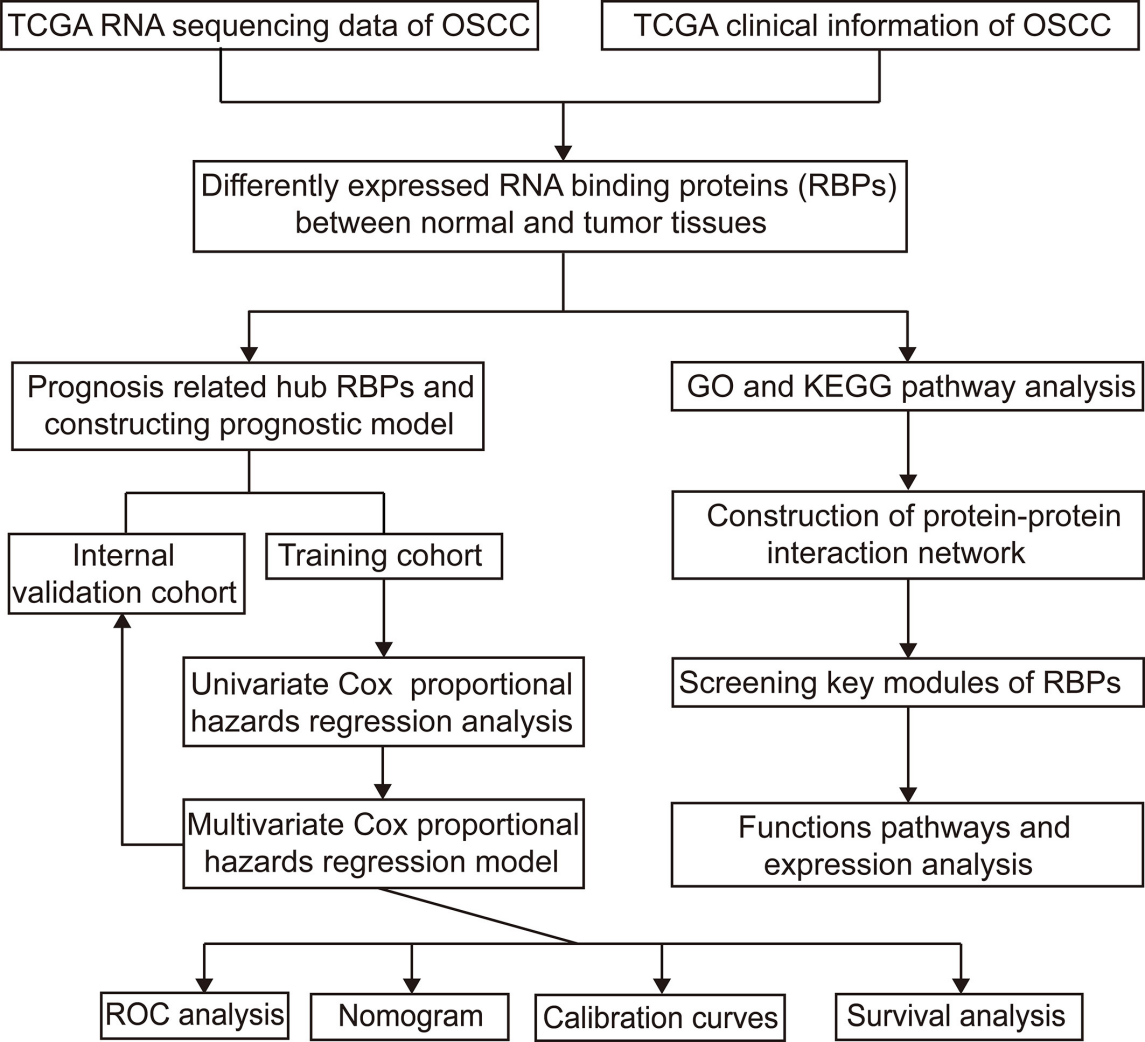
Study flowchart for analyzing RBPs in OSCC. OSCC, oral squamous cell carcinoma; RBPs, RNA binding proteins.

**Figure 2:**
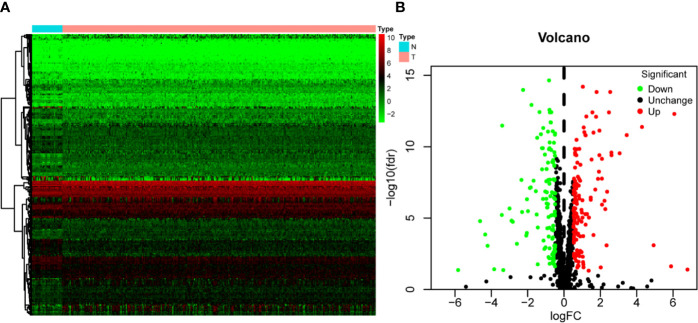
The differentially expressed RBPs in OSCC. **(A)** Hierarchical clustering of OSCC tissues and normal tissues by differentially expressed RBPs. The upper horizontal axis represents samples, and the left vertical axis represents clusters of RBPs. Red represents upregulated RBPs, and green represents downregulated RBPs. **(B)** Volcano plot of differentially expressed RBPs. The red dots represent upregulated RBPs, and the green dots represent downregulated RBPs. FC, Fold Change; fdr, false discovery rate.

**Figure 1** shows the workflow of this study. RNA expression data and corresponding clinical information were downloaded from TCGA. In total, 331 OSCC samples and 32 normal oral samples were analyzed. The Limma R software package was used to preprocess these data and to identify differentially expressed RBPs (11). A total of 1,542 RBPs were analyzed in this study, and 257 met our inclusion criteria (P<0.05, |log2FC)| >1.0), including 121 downregulated and 136 upregulated RBPs (**Figure 2**).

### Functional Enrichment Analysis of the Differentially Expressed RBPs

**Figure 3:**
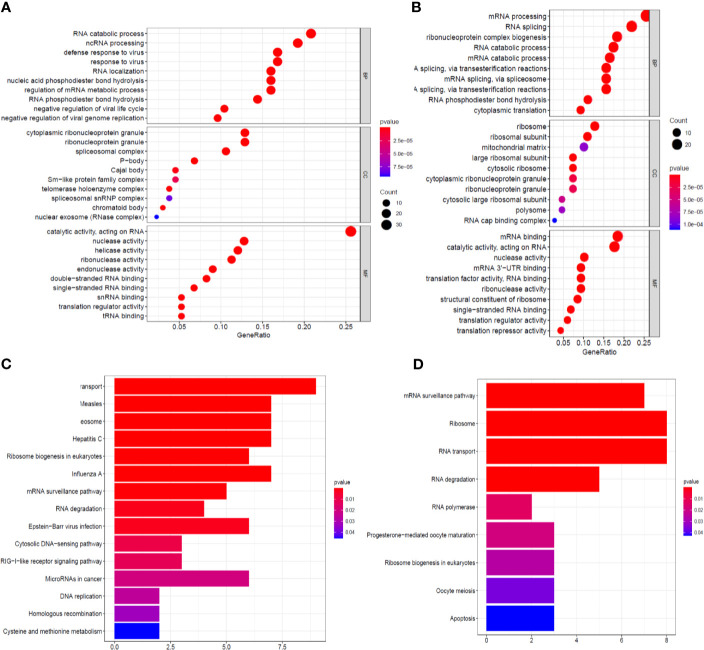
GO enrichment and KEGG pathway analyses of aberrantly expressed RBPs. Biological process, cellular components, and molecular function enrichment for up-expressed RBPs **(A)** and down-expressed RBPs **(B)**; KEGG pathway analysis for up-expressed RBPs **(C)** and down-expressed RBPs **(D)**.

The identified RBPs were divided into two groups, the upregulation group and the downregulation group, to explore their potential functions and mechanisms. The upregulated RBPs were significantly enriched in defense response to viruses and regulation of mRNA metabolic processes by GO and pathway analyses (**Supplementary Table 1** and **Figure 3A**), while the downregulated RBPs were considerably enriched in mRNA processing, RNA splicing, and RNA catabolic processes (**Supplementary Table 1** and **Figure 3B**). Additionally, the KEGG pathway analysis results showed that the upregulated RBPs were enriched in the RNA transport, spliceosome, and mRNA surveillance pathways (**Supplementary Table 1** and **Figure 3C**), while the downregulated RBPs were mainly enriched in the RNA transport, ribosome, and mRNA surveillance pathways (**Supplementary Table 1** and **Figure 3D**).

### PPI Network Construction and Key Module Screening

**Figure 4:**
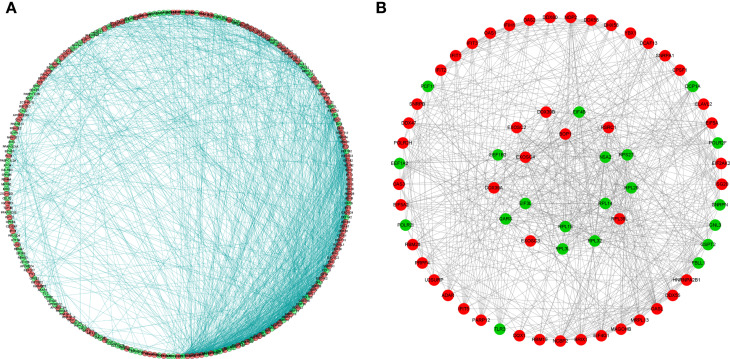
Protein-protein interaction network and modules analysis. **(A)** Protein-protein interaction network of differentially expressed RBPs; **(B)** Significant modules from PPI network. Green circles: down-expressed RBPs; red circles: up-expressed RBPs.

To better understand the potential roles of these identified RBPs in OSCC, we constructed a PPI network using the STRING database and Cytoscape software. This PPI network consists of 228 nodes and 1,384 edges in total (**Figure 4A**). We further built the coexpression network using the MCODE plug-in and identified the key modules (**Figure 4B**). Of these, the first essential module, module 1, contained 49 nodes and 316 edges; the second module, module 2, consisted of 8 nodes and 28 edges; module 3 consisted of 13 nodes and 43 edges; module 4 consisted of 6 nodes and 12 edges; the next module consisted of 4 nodes and 6 edges; and the last module consisted of 16 nodes and 29 edges (**Supplementary Figures 1A–F**). The functional analysis showed that the RBPs in these key modules were enriched in mitochondrial translation, mitochondrial gene expression, and cellular protein complex disassembly (**Supplementary Table 1**).

### Identification of Prognosis-Related RBPs

A total of 20 prognosis-associated candidate hub RBPs were identified by univariate Cox regression analysis (**Supplementary Table 2**). We further analyzed the candidate RBPs by multivariate Cox regression to determine their significance for prognosis. Ten hub RBPs—ZC3H12D, OAS2, INTS10, ACO1, PCBP4, RNASE3, PTGES3L-AARSD1, RNASE13, DDX4, and PCF11—were identified as independent predictors of OSCC (**Supplementary Table 3**). According to the expression profiles of 10 RBPs, cluster analysis was performed to analyze the 331 OSCC samples from the entire TCGA cohort, and two subtypes were determined, which demonstrates associated heterogeneity can be effectively identified by 10 RBPs model (**Supplementary Figure 2A**). Subsequently, the result of principal component analysis (PCA) indicates that 10 RBPs can distinguish OSCC patients in training cohort, validation cohort, and entire TCGA cohort (**Supplementary Figures 2B–D**).

### Prognosis-Related Genetic RS Model Construction and Validation

**Figure 5:**
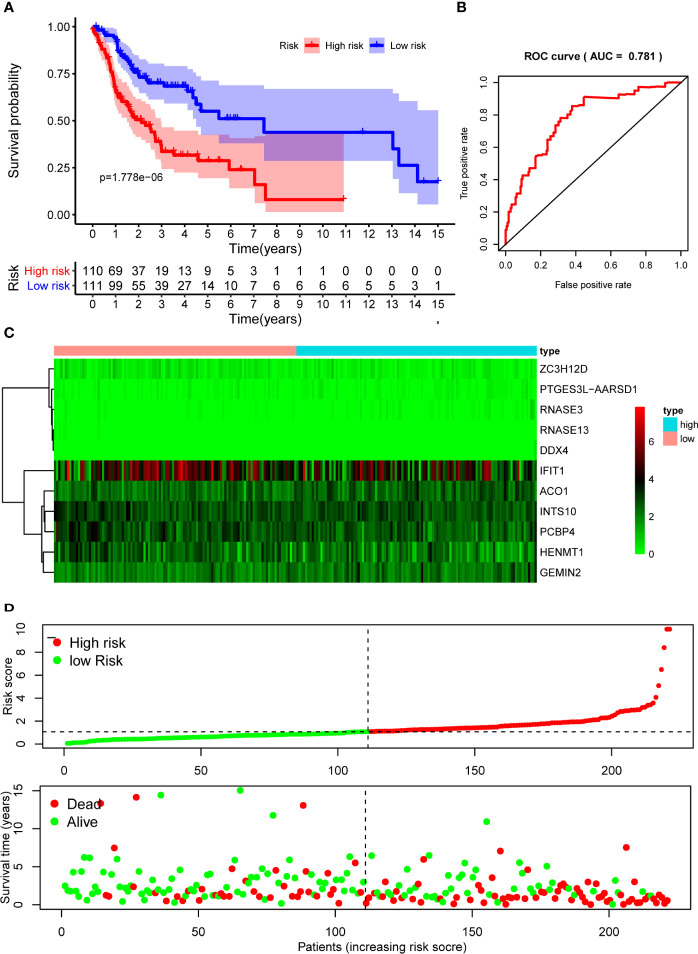
Risk score analysis of 10-gene prognostic model in the training cohort. **(A)** Survival curves for OS in low- and high-risk subgroups; **(B)** ROC curves for predicting OS based on risk score; **(C)** Expression heat map of 10 prognostic RBPs in low- and high-risk subgroups; **(D)** Risk score distribution and survival status for low- and high-risk subgroups. OS, overall survival; ROC, receiver operating characteristic.

**Figure 6:**
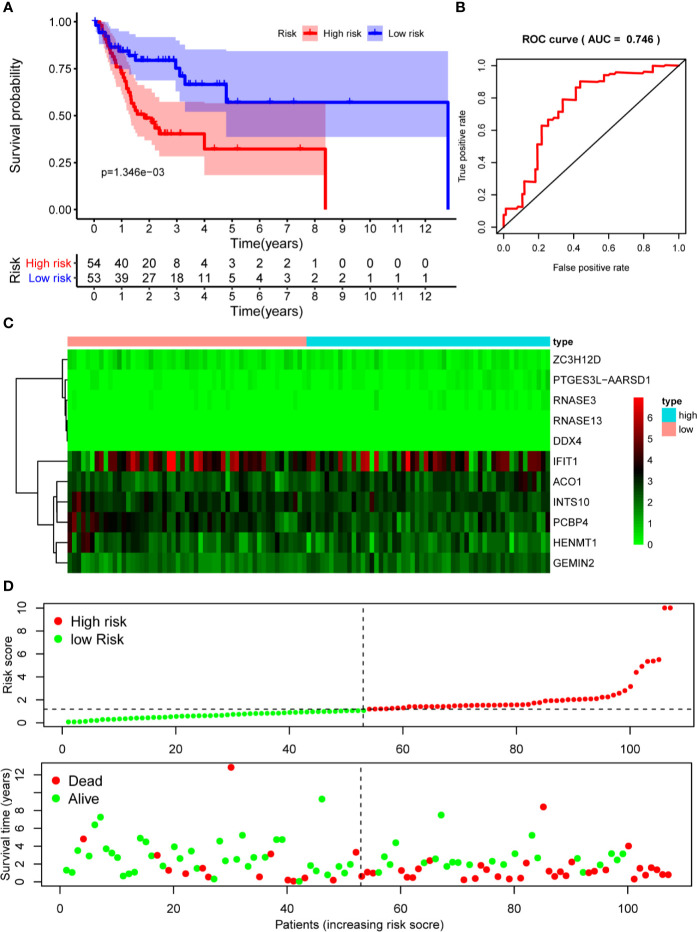
Risk score analysis of 10-gene prognostic model in the internal validation cohort. **(A)** Survival curves for OS in low- and high-risk subgroups; **(B)** ROC curves for predicting OS based on risk score; **(C)** Expression heat map of 10 prognostic RBPs in low- and high-risk subgroups; **(D)** Risk score distribution and survival status for low- and high-risk subgroups. OS, overall survival; ROC, receiver operating characteristic.

The 10 hub RBPs were used to build a prognostic model. To assess the predictive ability of this model, we further carried out an RS analysis in the training cohort. The RS of each patient was calculated according to the following formula: RS=ZC3H12D*-1.505+OAS2*-0.006+INTS10*-0.135+ACO1*0.108+PCBP4*-0.122+RNASE3*2.757+PTGES3L-AARSD1*2.827+RNASE13*-11.340+DDX4*8.283+PCF11*-0.204. A total of 221 OSCC patients were divided into low-risk and high-risk groups according to the median RS. The results showed that patients in the low-risk group had significantly longer OS times than those in the high-risk group (**Figure 5A**). Time-dependent ROC curve analysis was further conducted to estimate the prognostic ability of the identified RBPs. The area under the ROC curve (AUC) of the RS model was 0.781 (**Figure 5B**), which suggested that our model has high prognostic accuracy. The expression heat map of the 10 prognostic RBPs is illustrated in **Figure 5C**, and the RS and survival status for the low- and high-risk groups are displayed in **Figure 5D**. Moreover, the results from our internal validation cohort, using the same formula used to assess the predictive capability of the 10-gene prognostic model, showed that patients with lower RSs had better OS (**Figures 6A–D**). These results indicated that the prognostic model has significant predictive capability.

### Prognostic Value of Different Clinical Parameters

**Figure 7:**
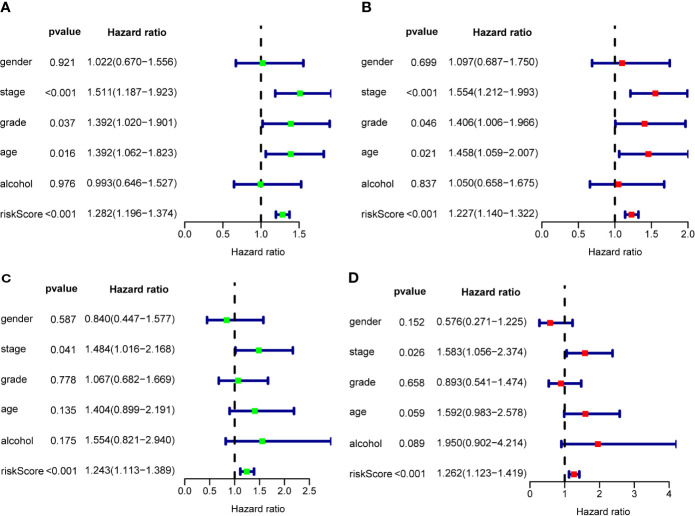
The prognostic value of different clinical parameters. Univariate Cox regression analysis and forest plots of the HR and 95% CIs in the training cohort **(A)** and internal validation cohort **(C)**; Multivariate Cox regression analysis and forest plots of the HR and 95% CIs in the training cohort **(B)** and internal validation cohort **(D)**.

To assess the prognostic significance of different clinical features, we performed a univariate Cox regression analysis of OSCC patients in the training set and internal validation set. The results showed that stage, grade, age, and RS were correlated with the OS of OSCC patients in both cohorts (P<0.05) (**Figures 7A, B**), whereas only stage and RS were independent prognostic factors for OS in the multiple regression analysis of both cohorts (P<0.05) (**Figures 7C, D**).

### Development and Validation of a Nomogram Based on the Hub RBPs

**Figure 8:**
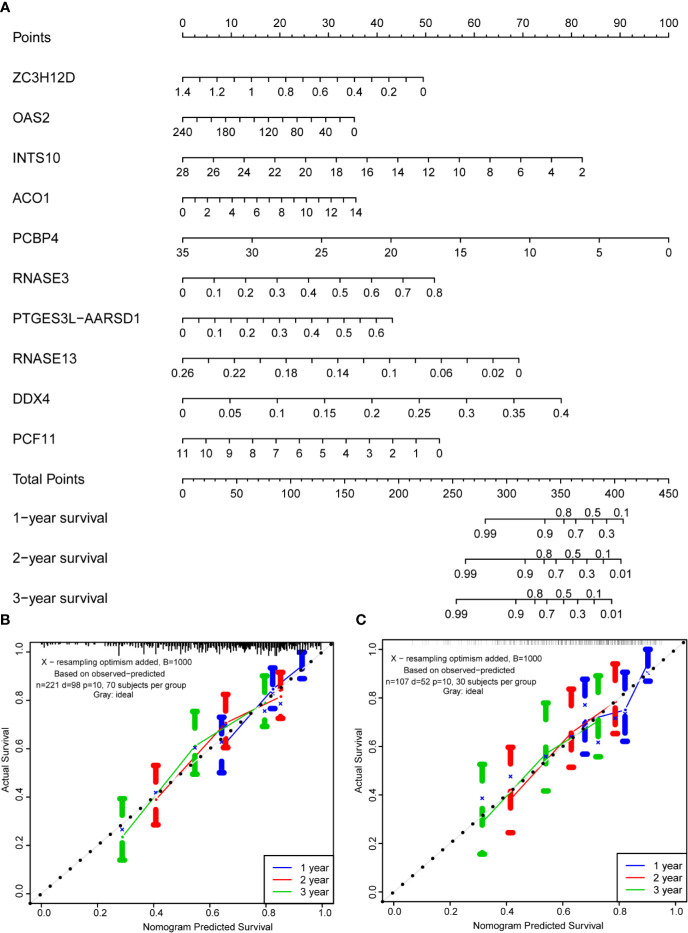
Prognostic nomogram based on hub RBPs and calibration plots. **(A)** Nomogram for predicting the 1-, 2-, and 3-year OS in the training cohort. All the points identified on the top scale for each factor were added to generate a total score. The total points projected on the bottom scale were used to determine the probabilities of 1-, 2-, and 3-year OS for each patient. Nomograms for predicting the 1-, 2-, and 3-year OS probabilities of OSCC patients in the training cohort **(B)** and internal validation cohort **(C)**.

Based on the multivariate analysis results, a nomogram was established to predict the 1-, 2-, and 3-year OS (**Figure 8A**). We list the points for each independent prognostic factor in the nomogram and summed them to obtain the total point value, which was drawn on the bottom scale. We plotted the calibration curves, which indicated no apparent deviation from the ideal line, indicating our model might be effectively used to predict outcomes and survival rates of OSCC patients (**Figures 8B, C**).

Next, the protein expression pattern of 10 RBPs in the prognosis model were analyzed in the tissues of OSCC patients from Sun Yat-Sen Memorial Hospital, Sun Yat-Sen University. The results demonstrated that the expression of the ZC3H12D, OAS2, INTS10, PCBP4, RNASE13, PCF11 protein were low expressed in OSCC tissues than that in normal tissues. ACO1, DDX4, PTGES3L-AARSD1, RNASE3 were high expressed in OSCC tissues than that in normal tissues (**Supplementary Figure 3**).

## Discussion

The survival rate of OSCC patients remains low because there are limited prognostic factors that can provide effective targeted therapies and precisely predict outcomes (9). Previous studies have demonstrated that RBPs, which regulate oncogene expression at the posttranscriptional level, significantly affect tumorigenesis and progression, indicating that RBPs are potential cancer biomarkers (27, 28). In fact, several RBPs have been identified to predict OS in cancer. Zhao et al. identified a novel extra-ribosomal role of RPS3 as a potential therapeutic target in HCC *via* the posttranscriptional regulation of SIRT1 expression (29). Soni et al. highlighted that MK2, as the master regulator of RBPs, plays an important role in the regulation of transcript stability and tumor progression, as well as the possibility of the use of MK2 as a therapeutic target in tumor management (30). However, the role of RBPs in OSCC is not well understood, even though studies have focused on the functions of RBPs in head and neck carcinoma. Berggen et al. revealed that MK2 could be a potential prognostic biomarker for head and neck cancer and that MK2 pathway activation can mediate radiation resistance in HNSCC (31). Jiang et al. indicated that LINC00460 is a promising candidate prognostic predictor for HNSCC because it facilitates the entry of an RBP called PRDX1 into the nucleus and promotes the epithelial-mesenchymal transition (EMT) in HNSCC cells (32). However, most of these studies analyzed the functions of single RBPs, while prognostic markers including several RBPs should be collectively investigated (33).

In the present study, we identified dysregulated RBPs in OSCC based on TCGA database and systematically analyzed the related biological pathways of these RBPs. We further constructed a PPI network to identify the key modules that may have significant impact on the prognosis of OSCC. Subsequently, we performed Cox regression analysis and identified 10 prognosis-related RBPs. Moreover, we conducted RS analysis of this 10-gene prognostic model in the training and validation cohorts to explore the potential roles of these 10 hub RBPs. The model exhibited good predictive capability in both cohorts. Finally, we built and validated a nomogram to predict OSCC prognosis based on these hub RBPs. Our findings may provide novel insights into the prognosis of OSCC patients.

The biological function and pathway enrichment analyses showed that the downregulated RBPs were markedly enriched in the mRNA processing, RNA splicing, RNA catabolic process, RNA transport, ribosome, and mRNA surveillance pathways. Upregulated RBPs were significantly enriched in the defense response to viruses, response to viruses, regulation of mRNA metabolic process, RNA transport, RNA degradation, spliceosome, and mRNA surveillance pathways. Many studies have revealed the role of metabolism, RNA degradation, and RNA transport in OSCC (34–36). Human papillomavirus (HPV) is closely associated with OSCC (37). HPV infection significantly increases the number of binding sites of RBPs and subsequently upregulates a group of oncogenic genes by absorbing RBPs (38). A previous study showed that RBPs can regulate mRNA stability or translational efficiency *via* ribosomes and are involved in various diseases (39). These reports indicated that RBPs may affect the biological behavior of OSCC by interacting with components related to HPV or regulating the multiple biological processes of OSCC cells.

We identified 10 hub RBPs by constructing a PPI network and performing Cox regression analysis, inducing ZC3H12D, OAS2, INTS10, ACO1, PCBP4, RNASE3, PTGES3L-AARSD1, RNASE13, DDX4, and PCF11. Most of these RBPs are associated with tumorigenesis and cancer progression, including OAS2 (40), INTS10 (41), ACO1 (42), PCBP4 (43), DDX4 (44), and PCF11 (45). Of these, OAS2, an interferon (IFN)-induced antiviral enzyme, has been reported as an OSCC-related gene. Similarly, Wang et al. analyzed three microarray datasets and found that OAS2 expression was closely related to the survival rate of patients with oral cancer (46), which was consistent with our results. Interestingly, the IFN system is a central and powerful host first-line antiviral defense strategy that is inhibited by HPV16 in HNSCC (47). A substantial OAS2 level can activate the IFN signaling pathway and in turn produce a T-cell response that is effective in clearing HPV infection (48). Thus, OAS2 may serve as a key regulator and may provide rational insight into immunotherapy for HPV-associated OSCC. However, the detailed mechanisms underlying these hub RBPs in OSCC are still unexplored, and further in-depth studies to discover their potential roles would be valuable.

Subsequently, we established a risk model including the 10 hub RBPs. The ROC analysis revealed that these 10 genes had a good diagnostic capability to identify OSCC patients with a poor prognosis. Next, a prognostic nomogram was built to predict OSCC patients’ survival. Finally, calibration curves were plotted to detect the prognostic value of the 10 RBP coding genes, and the results indicated that the nomogram-predicted outcomes were consistent with the actual survival rates.

To sum up, all the evidence suggested that our prognostic model including 10 RBPs has a certain value for the treatment decisions of OSCC patients. Nonetheless, there are still several limitations in our study. The main one is the lack of clinical data. Survival analysis included only basic clinical factors which are obtained from TCGA database. However, as we have known, other factors, such as the HPV status of patients, may affect the reliability of the Cox regression analysis. In addition, the results of functional enrichment analysis in this study have suggested that some RBPs may affect the outcome of OSCC patients through interactions with virus. If we have more clinical information, we were able to have a better understanding of combinations between RBPs and HPV infection. Another limitation lies in the expression of all genes from TCGA cohort was detected in an OSCC tissue sample from one patient. But OSCC is an extremely heterogeneous tumor at both genetic and molecular levels. In the future, clinical trials are needed. More analysis in multiple OSCC specimens from one patient should be applied to confirm our results. Besides, cooperation with other hospitals is also needed to enlarge the clinical sample size for model validation.

## Conclusion

We systematically explored the prognostic value of differentially expressed RBPs by performing a series of bioinformatics analyses on OSCC. A prognostic model of the 10 hub RBPs was constructed and further validated. Our results were used to establish a novel prognostic model for OSCC and will contribute new candidate markers to aid in therapeutic decision-making in the future.

## Author’s Note

This manuscript has been released as a preprint at ResearchSquare.

## Data Availability Statement

The datasets presented in this study can be found in online repositories. The names of the repository/repositories and accession number(s) can be found in the article/**Supplementary Material**.

## Ethics Statement

For TCGA database, approval of the institutional ethics committee was not necessary because the data were collected from publicly available online databases. For external validation cohort, all procedures were approved by the institutional ethics committee of Sun Yat-Sen Memorial Hospital, and informed consent was obtained from all patients.

## Author Contributions

Conception and design: YLu and YYa. Development of methodology: YLu and YYa. Analysis and interpretation of data (e.g., statistical analysis, bioinformatics analysis): YLu, YYa, BL, ML, and YLi. Writing, review, and/or revision of the manuscript: YLu, YYa, BL, ML, YLi, YYe, WC, JJ, JL, and SC. Administrative, technical, or material support (i.e., reporting or organizing data): YLu, BL, ML, YLi, JL, JJ, and SC. Study supervision: JL, JJ, and SC. All authors contributed to the article and approved the submitted version.

## Funding 

This work was supported by the National Natural Science Foundation of China (NSFC; 81900959 to YLu, 81802700 to YLi, 82072990 and 81872194 to JL).

## Conflict of Interest

The authors declare that the research was conducted in the absence of any commercial or financial relationships that could be construed as a potential conflict of interest.

The reviewer WW declared a shared affiliation, with no collaboration, with the authors to the handling editor at the time of review.

## Publisher’s Note

All claims expressed in this article are solely those of the authors and do not necessarily represent those of their affiliated organizations, or those of the publisher, the editors and the reviewers. Any product that may be evaluated in this article, or claim that may be made by its manufacturer, is not guaranteed or endorsed by the publisher.

## Supplementary Material

Supplementary Figure 1 Modules analysis of protein-protein interaction (PPI) network. **(A)** module 1 from PPI network; **(B)** module 2 from PPI network; **(C)** module 3 from PPI network; **(D)** module 4 from PPI network; **(E)** module 5 from PPI network; **(F)** module 6 from PPI network. Green circles: down-expressed RBPs; red circles: up-expressed RBPs. Related to **Figure 4**.

Supplementary Figure 2 Cluster analysis based on 10 RBPs. **(A)** Cluster analysis indicated that 331 OSCC samples in TCGA can be divided into two groups. Principal component analysis was performed on the basis of cluster analysis in training cohort **(B)**, validation cohort **(C)**, and entire TCGA cohort **(D)**.

Supplementary Figure 3 Immunohistochemistry results in the prognosis-related 10 RBPs. Scale bar: 50 μm.
