# Supplementary figures and images for: A Novel Prognostic Model for Oral Squamous Cell Carcinoma: The Functions and Prognostic Values of RNA-Binding Proteins
Source: Front Oncol. 2021 Jul 30;11:592614. doi: 10.3389/fonc.2021.592614 (PMC8362834; doi:10.3389/fonc.2021.592614)

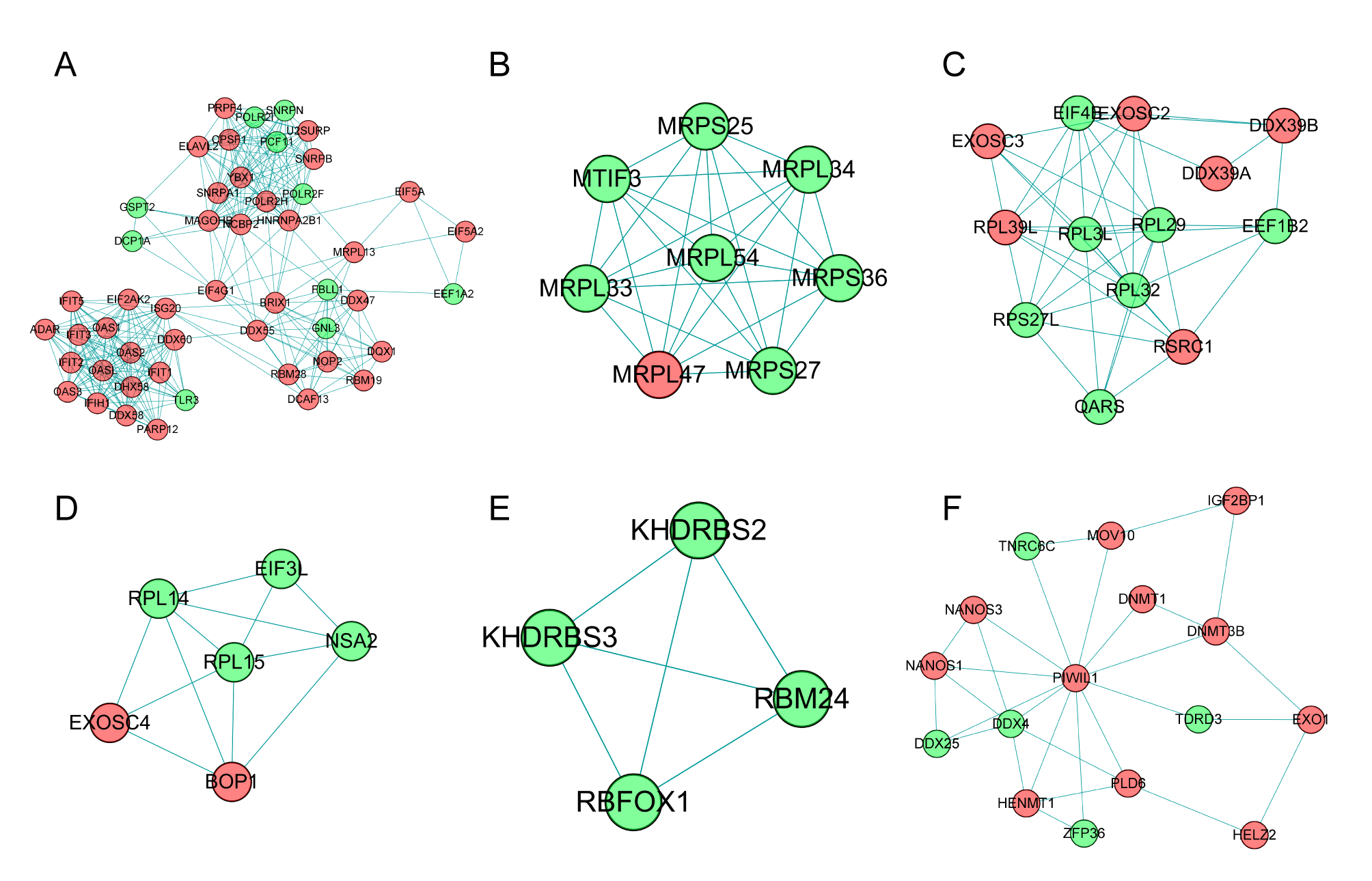

Supplement: Supplementary Figure 1 — Modules analysis of protein-protein interaction (PPI) network. (A) module 1 from PPI network; (B) module 2 from PPI network; (C) module 3 from PPI network; (D) module 4 from PPI network; (E) module 5 from PPI network; (F) module 6 from PPI network. Green circles: down-expressed RBPs; red circles: up-expressed RBPs. Related to Figure 4. [file Image_1.tif]

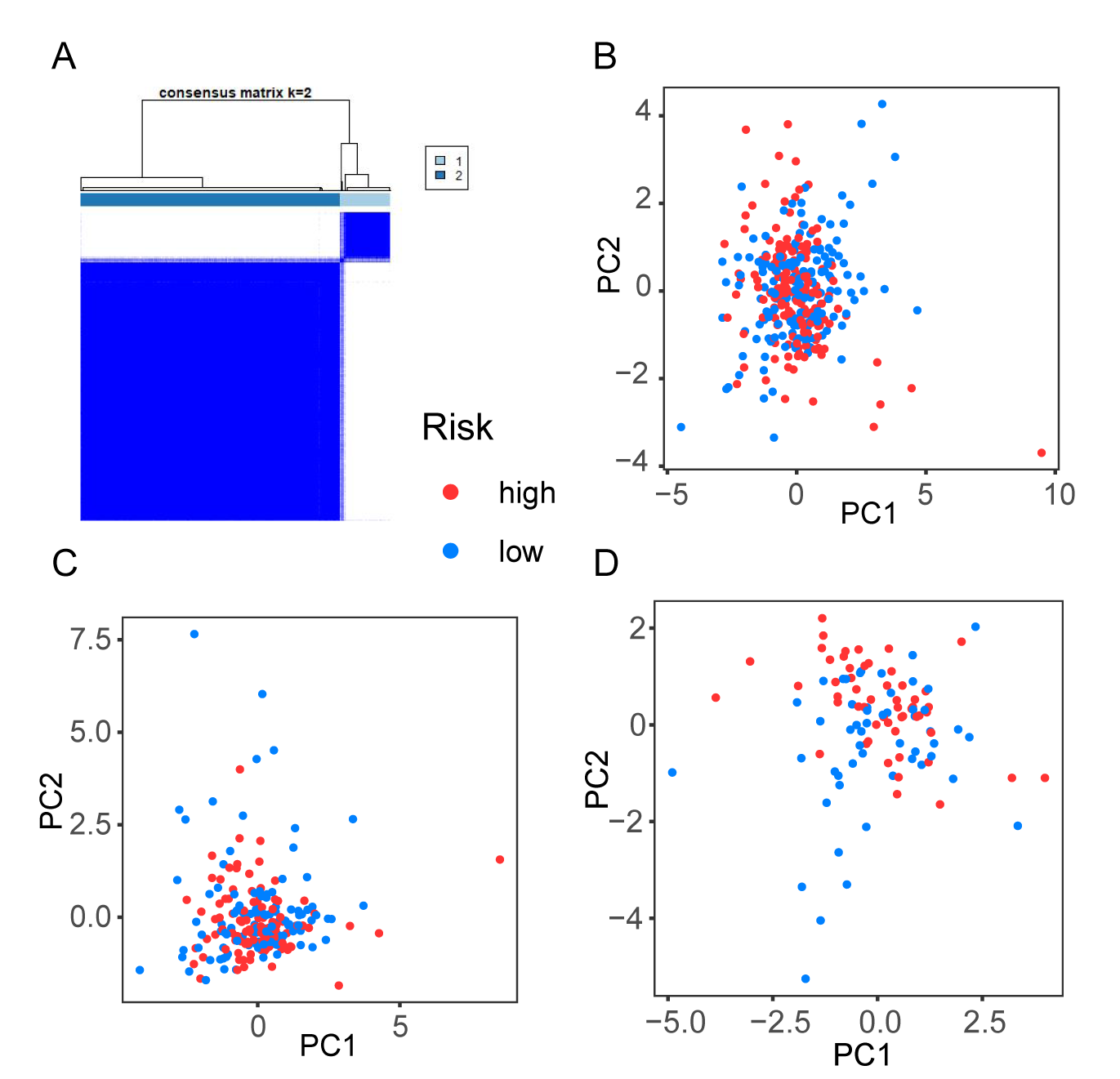

Supplement: Supplementary Figure 2 — Cluster analysis based on 10 RBPs. (A) Cluster analysis indicated that 331 OSCC samples in TCGA can be divided into two groups. Principal component analysis was performed on the basis of cluster analysis in training cohort (B), validation cohort (C), and entire TCGA cohort (D). [file Image_2.tif]

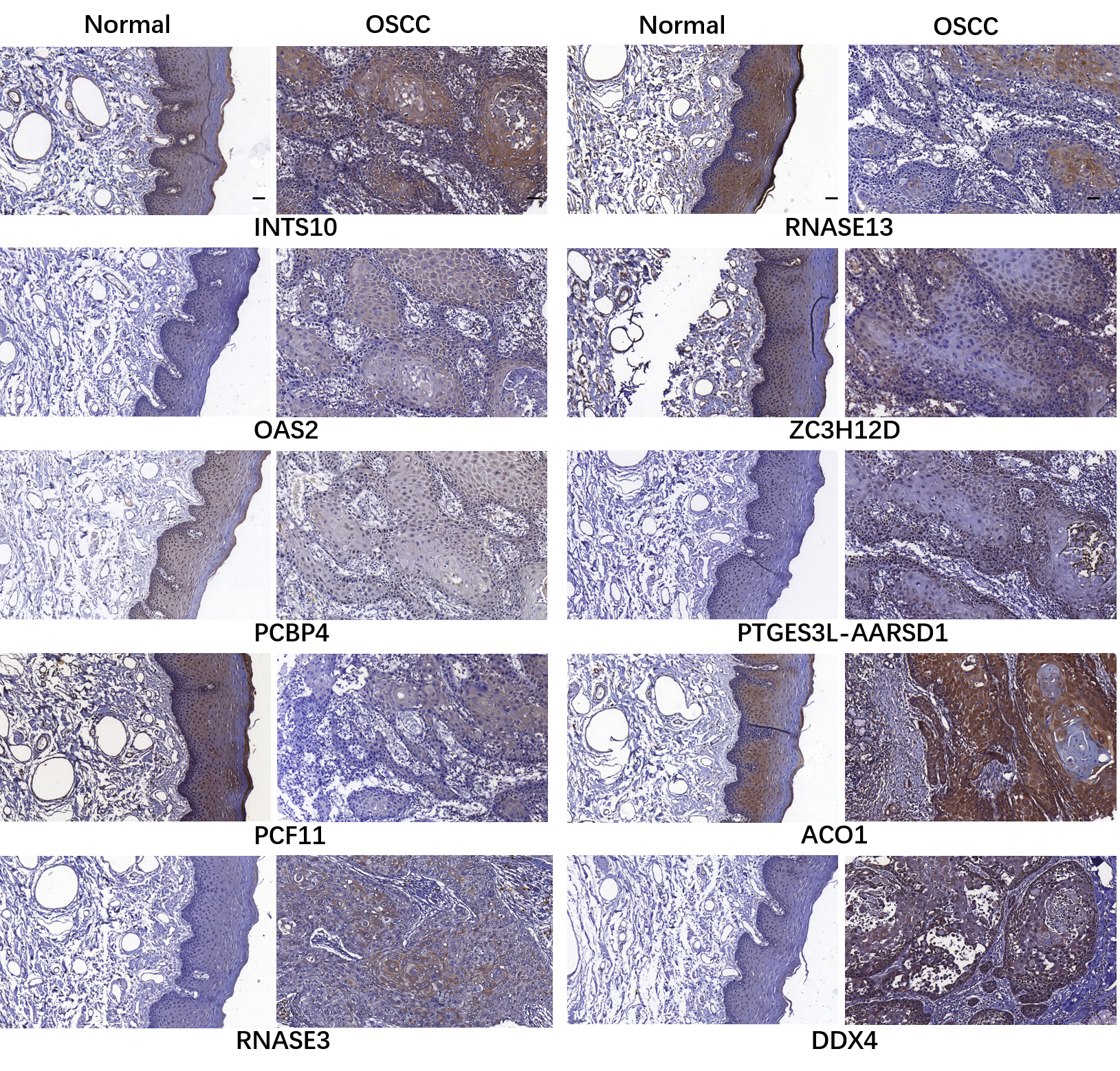

Supplement: Supplementary Figure 3 — Immunohistochemistry results in the prognosis-related 10 RBPs. Scale bar: 50 μm. [file Image_3.tif]
