# Supplementary material for: A Novel Prognostic Model for Oral Squamous Cell Carcinoma: The Functions and Prognostic Values of RNA-Binding Proteins
Source: Front Oncol. 2021 Jul 30;11:592614. doi: 10.3389/fonc.2021.592614 (PMC8362834; doi:10.3389/fonc.2021.592614)
Supplement: Supplementary file 4 [file Table_1.docx]

Supplementary Table 1 GO and KEGG pathway analysis results for differentially expressed RBPs in vital modules.

| Module | Item | Description | P-value |
| --- | --- | --- | --- |
| Module 1 | BP | defense response to virus | 4.15E-19 |
|  |  | mRNA processing | 1.25E-17 |
|  |  | RNA splicing | 2.60E-17 |
|  | CC | spliceosomal complex | 5.39E-11 |
|  |  | catalytic step 2 spliceosome | 6.57E-06 |
|  |  | RNA polymerase II, core complex | 1.34E-05 |
|  | MF | double-stranded RNA binding | 2.03E-15 |
|  |  | snRNA binding | 1.05E-09 |
|  |  | nucleotidyltransferase activity | 1.27E-07 |
|  | KEGG | Spliceosome | 1.51E-05 |
|  |  | mRNA surveillance pathway | 1.90E-05 |
|  |  | Cytosolic DNA-sensing pathway | 8.05E-05 |
| Module 2 | BP | mitochondrial translation | 6.95E-18 |
|  |  | mitochondrial gene expression | 2.94E-17 |
|  |  | mitochondrial translational elongation | 4.40E-16 |
|  | CC | organellar ribosome | 2.05E-13 |
|  |  | mitochondrial ribosome | 2.05E-13 |
|  |  | ribosome | 4.41E-13 |
|  | MF | structural constituent of ribosome | 3.45E-09 |
|  |  | ribosome binding | 0.000247717 |
|  |  | ribonucleoprotein complex binding | 0.001264043 |
|  | KEGG | Ribosome | 0.000383794 |
| Module 3 | BP | ribonucleoprotein complex biogenesis | 2.96E-10 |
|  |  | ribosome biogenesis | 6.85E-08 |
|  |  | nuclear-transcribed mRNA catabolic process | 2.64E-07 |
|  | CC | cytosolic ribosome | 1.40E-08 |
|  |  | cytosolic large ribosomal subunit | 1.12E-07 |
|  |  | ribosomal subunit | 1.34E-07 |
|  | MF | structural constituent of ribosome | 7.64E-08 |
|  |  | catalytic activity, acting on RNA | 4.85E-06 |
|  |  | helicase activity | 0.000167686 |
|  | KEGG | Ribosome | 1.68E-05 |
|  |  | RNA degradation | 0.003281941 |
|  |  | RNA transport | 0.016180765 |
| Module 4 | BP | rRNA processing | 4.35E-09 |
|  |  | rRNA metabolic process | 7.98E-09 |
|  |  | ribosome biogenesis | 1.41E-08 |
|  | CC | preribosome, large subunit precursor | 2.57E-05 |
|  |  | cytosolic large ribosomal subunit | 0.000193649 |
|  |  | preribosome | 0.000248415 |
|  | MF | structural constituent of ribosome | 0.001239996 |
|  |  | cadherin binding | 0.004853694 |
|  |  | AU-rich element binding | 0.008518805 |
|  | KEGG | Ribosome | 0.001136486 |
|  |  | RNA degradation | 0.029192513 |
| Module 5 | BP | regulation of mRNA metabolic process | 7.04E-08 |
|  |  | regulation of mRNA splicing, via spliceosome | 3.44E-07 |
|  |  | regulation of mRNA processing | 1.13E-06 |
|  | CC | trans-Golgi network | 0.042521297 |
|  |  | SH3 domain binding | 0.000299216 |
|  |  | mRNA binding | 0.003031579 |
|  | MF | poly(A) binding | 0.003642465 |
| Module 6 | BP | regulation of nuclear-transcribed mRNA catabolic process, deadenylation-dependent decay | 8.14E-10 |
|  |  | positive regulation of nuclear-transcribed mRNA catabolic process, deadenylation-dependent decay | 8.14E-10 |
|  |  | piRNA metabolic process | 1.37E-09 |
|  | CC | cytoplasmic ribonucleoprotein granule | 1.23E-12 |
|  |  | ribonucleoprotein granule | 1.78E-12 |
|  |  | P granule | 3.47E-07 |
|  | MF | helicase activity | 8.85E-06 |
|  |  | catalytic activity, acting on RNA | 1.56E-05 |
|  |  | endonuclease activity | 0.000231732 |
|  | KEGG | Cysteine and methionine metabolism | 0.00035966 |
|  |  | MicroRNAs in cancer | 0.000535848 |
|  |  | Mismatch repair | 0.014225399 |

Supplementary Table 2 Prognosis-associated hub RBPs identified by univariate Cox regression analysis.

| RBP name | HR | Lower 95% CI | Upper 95% CI | P-value |
| --- | --- | --- | --- | --- |
| ZNFX1 | 0.976 | 0.954 | 0.998 | 0.037 |
| MKRN2 | 0.933 | 0.872 | 0.997 | 0.041 |
| MKRN3 | 1.760 | 1.164 | 2.663 | 0.007 |
| ZC3H12D | 0.290 | 0.115 | 0.730 | 0.009 |
| HENMT1 | 0.926 | 0.864 | 0.992 | 0.029 |
| QARS | 0.983 | 0.967 | 1.000 | 0.046 |
| PARP12 | 0.975 | 0.952 | 0.998 | 0.036 |
| OAS2 | 0.995 | 0.991 | 1.000 | 0.042 |
| INTS10 | 0.930 | 0.866 | 0.998 | 0.044 |
| ACO1 | 1.085 | 1.016 | 1.158 | 0.014 |
| PCBP4 | 0.942 | 0.893 | 0.994 | 0.028 |
| RNASE3 | 8.469 | 1.341 | 53.469 | 0.023 |
| PTGES3L-AARSD1 | 5.343 | 1.206 | 23.675 | 0.027 |
| RNASE13 | 0.000 | 0.000 | 0.257 | 0.025 |
| DDX4 | 225.948 | 1.251 | 40814.968 | 0.041 |
| PCF11 | 0.837 | 0.724 | 0.969 | 0.017 |
| CELF2 | 0.877 | 0.780 | 0.987 | 0.029 |
| SECISBP2L | 0.890 | 0.794 | 0.997 | 0.044 |
| GEMIN2 | 1.081 | 1.004 | 1.164 | 0.039 |
| IFIT1 | 0.993 | 0.987 | 0.999 | 0.035 |

Supplementary Table 3 Ten prognosis-associated hub RBPs identified by multivariate Cox regression analysis.

| RBP name | coef | HR | Lower 95% CI | Upper 95% CI | P-value |
| --- | --- | --- | --- | --- | --- |
| ZC3H12D | -1.505 | 0.222 | 0.059 | 0.834 | 0.026 |
| OAS2 | -0.006 | 0.994 | 0.987 | 1.000 | 0.066 |
| INTS10 | -0.135 | 0.874 | 0.783 | 0.976 | 0.017 |
| ACO1 | 0.108 | 1.114 | 1.026 | 1.211 | 0.010 |
| PCBP4 | -0.122 | 0.885 | 0.822 | 0.954 | 0.001 |
| RNASE3 | 2.757 | 15.758 | 1.409 | 176.263 | 0.025 |
| PTGES3L-AARSD1` | 2.827 | 16.896 | 1.728 | 165.251 | 0.015 |
| RNASE13 | -11.340 | 0.000 | 0.000 | 22.183 | 0.124 |
| DDX4 | 8.283 | 3955.314 | 15.357 | 1018717.438 | 0.003 |
| PCF11 | -0.204 | 0.815 | 0.662 | 1.003 | 0.054 |
